# Rare Hepatic Arterial Anatomic Variants in Patients Requiring Pancreatoduodenectomy and Review of the Literature

**DOI:** 10.1155/2012/953195

**Published:** 2012-07-30

**Authors:** Smita Ramanadham, Seth M. Toomay, Adam C. Yopp, Glen C. Balch, Rohit Sharma, Roderich E. Schwarz, John C. Mansour

**Affiliations:** ^1^Division of Surgical Oncology, Department of Surgery, University of Texas Southwestern Medical Center, Dallas, TX 75390-9155, USA; ^2^Division of Interventional Radiology, Department of Radiology, University of Texas Southwestern Medical Center, Dallas, TX 75390-8896, USA

## Abstract

Normal hepatic arterial anatomy occurs in approximately 50–80% of cases; for the remaining cases, multiple variations have been described. Knowledge of these anomalies is especially important in hepatobiliary and pancreatic surgery in order to avoid unnecessary complications. We describe two cases of patients undergoing pancreatoduodenectomy for abnormalities in the head of the pancreas. Preoperative contrast-enhanced cross-sectional imaging demonstrated relevant, rare hepatic arterial variants: (1) a completely replaced hepatic arterial system with a gastroduodenal artery (GDA) arising directly from the celiac axis and (2) a replaced right hepatic artery originating from the superior mesenteric artery and traveling anterior to the pancreatic uncinate process and head. These findings were confirmed during pancreatoduodenectomy. Both of these variants have been rarely described with an incidence of <1.0%. In the present paper, we describe the hepatic arterial anomalies commonly encountered and clarify the important details associated with these variants as they pertain to pancreatoduodenectomy.

## 1. Introduction

The first description of aberrant hepatic arterial anatomy was published in 1756. Michels' autopsy series of 200 dissections provided a classification scheme later updated in 1994 by Hiatt (Tables 1 and 2) [1, 2]. Normally the common hepatic artery arises from the celiac axis with a rightward horizontal course along the upper border of the pancreas to the pylorus. The origin of the common hepatic artery (CHA) lies anterior to the main portal vein and branches into the gastroduodenal artery (GDA) and the proper hepatic artery (PHA). The PHA divides into left and right hepatic arterial branches. Ligation of the gastroduodenal artery is a critical step during pancreatoduodenectomy, and the location of this artery is often excluded in descriptions of these variants.

In approximately 10% of patients, the right hepatic artery originates not from the common hepatic artery but from the superior mesenteric artery. When this occurs, the replaced right hepatic artery typically travels cephalad to the uncinate process.

In this paper, we describe two patients with hepatic arterial anatomic variants: (1) a completely replaced hepatic arterial system with a gastroduodenal artery (GDA) arising directly from the celiac axis and the RHA arising from the SMA and (2) a replaced right hepatic artery originating from the superior mesenteric artery and traveling anterior to the pancreatic head. We discuss the importance of an awareness of these arterial variants and implications of operative management.

## 2. Case Reports

**Case 1 (Completely Replaced Hepatic Arterial System)**

**Figure 1:**
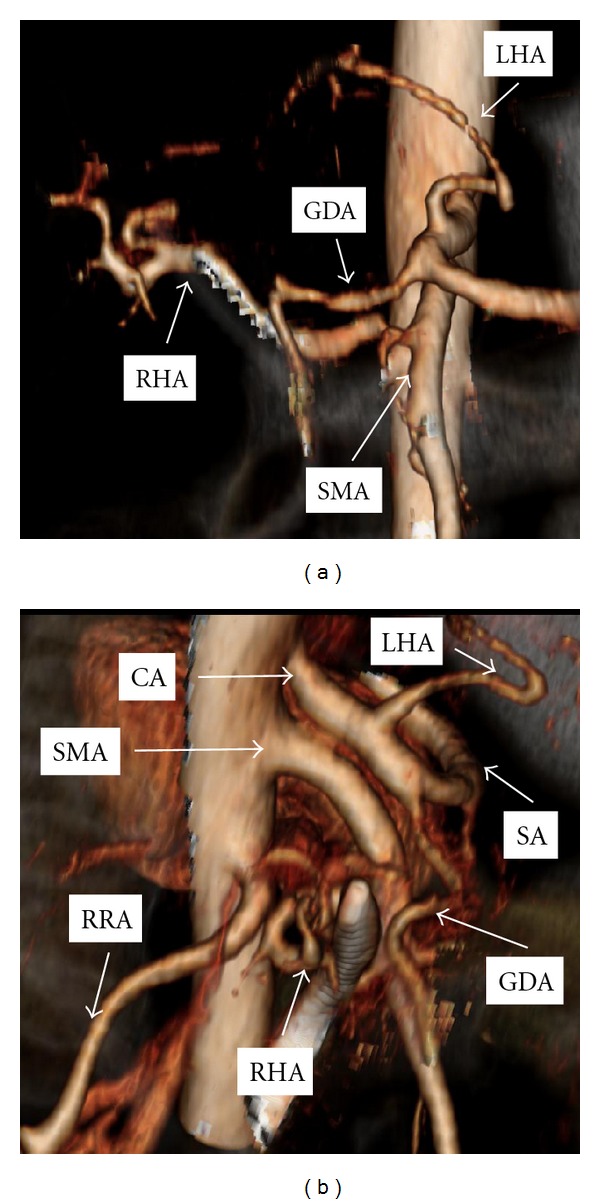
Coronal (a) and lateral (b) computed tomography reconstruction of mesenteric arterial vasculature in Case 1. Structure overlying right hepatic artery represents common bile duct stent. GDA: gastroduodenal artery, LHA: left hepatic artery, RHA: right hepatic artery, SMA: superior mesenteric artery, CA: celiac axis, RRA: right renal artery, SA: splenic artery.

A 62-year-old woman presented with right upper quadrant pain and jaundice. Endoscopic retrograde cholangiopancreatography (ERCP) revealed a common bile duct stricture and atypical cells, and a stent was placed. CT scan demonstrated a GDA arising directly from the celiac axis. In addition, a retroportal replaced RHA originated from the SMA and a replaced LHA originated from the left gastric artery (Figure 1).

Intraoperatively, a mass was appreciated near the upper portion of the pancreatic head. Aberrant arterial anatomy as described above was confirmed. We ligated and divided the GDA with no decrement in the palpable hepatic arterial pulse at the hilum or umbilical fissure and continued with the pancreatoduodenectomy. She experienced no postoperative adverse events, and pathologic evaluation showed a T3N1M0 moderately differentiated pancreatic ductal adenocarcinoma resected with negative margins.

**Case 2 (Replaced Right Hepatic Artery Embedded in the Pancreas)**

**Figure 2:**
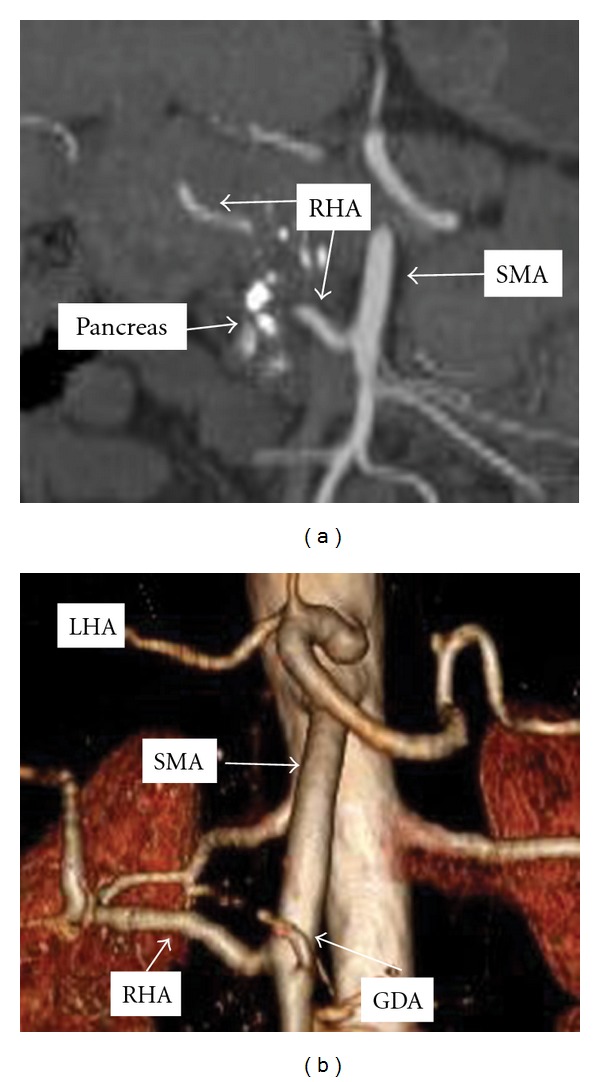
Coronal computed tomography reconstruction of mesenteric arterial vasculature in Case 2. GDA: gastroduodenal artery, LHA: left hepatic artery, RHA: right hepatic artery, SMA: superior mesenteric artery, Pancreas: calcified head of the pancreas.

A 55-year-old woman presented with a several-year history of recalcitrant chronic pancreatitis. CT scan demonstrated focal chronic pancreatitis in the head of the pancreas with a spared distal gland. CT arteriography showed a replaced right hepatic artery originating from the SMA and traveling anterior to the head of the pancreas just lateral to the neck of the pancreas (Figure 2). In addition, the GDA arose from the SMA caudad to the pancreas.

**Figure 3:**
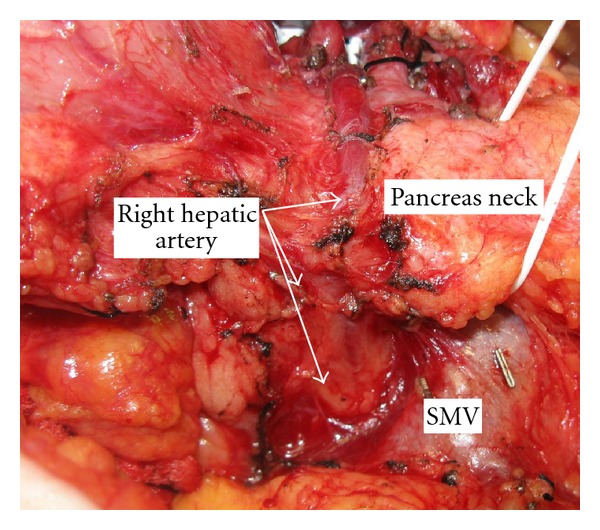
Intraoperative photograph of pancreas head in Case 2 with replaced right hepatic artery embedded in the anterior face of the pancreas.

Intraoperatively, no right-sided hepatic arterial system was identified from the common hepatic artery. After division of the pancreatic neck anterior to the superior mesenteric vein, mobilization of the pancreas head and uncinate process exposed the replaced right hepatic artery embedded in the anterior face of the pancreas gland (Figure 3). We divided the right hepatic artery at its origin from the SMA. She did well postoperatively and pathologic evaluation was consistent with chronic focal pancreatitis without malignancy.

## 3. Discussion

The most common variations include a replaced right hepatic artery (RHA) arising from the SMA in 10–15% of cases and a left hepatic artery originating from the left gastric artery in 3–10% of patients (Tables 1 and 2) [1, 2]. Although the hepatic artery supplies only 25–30% of the blood supply to the liver and can often be ligated, we prefer to spare the primary blood supply to the proximal bile duct. Ligation of the hepatic artery can result in ischemic biliary injury and breakdown of the biliary-enteric anastomosis [3]. Surgeons must have a comprehensive understanding of not only standard hepatobiliary arterial anatomy but also its variants in order to avoid potential complications.

Much of the information known about the hepatic arterial supply is derived from the radiology and anatomy literature. One such study describes the hepatic artery anatomy of 5002 patients undergoing CT and digital subtraction angiography. Normal anatomy was found in 89.1% of patients. 1.10% of patients had an absent CHA with the GDA originating separately [4]. A second study evaluated variants seen during digital subtraction angiography among 600 patients [5]. Two patients had a GDA as a branch of the celiac axis with the PHA as the first branch of the SMA. Neither study describes a replaced right hepatic artery running along the anterior aspect of the pancreas head, although this variant has been described in isolated reports [6, 7]. 

When encountering a replaced right hepatic artery, the surgeon can usually preserve the vessel. If the vessel runs through the pancreas, this may not be an option, and the artery may need to be sacrificed. Arterial reconstruction should be strongly considered in the setting of preoperative hyperbilirubinemia or simultaneous vein resection and reconstruction.

In summary, we have described a rare case of a GDA originating from the celiac axis in combination with a replaced hepatic arterial system. In addition, we describe a case of a replaced right hepatic artery coursing through pancreatic parenchyma with a completely replaced hepatic arterial system and GDA originating from the SMA. When performing hepatobiliary and pancreatic operations, especially pancreatoduodenectomy, it is vital to have a thorough knowledge of such anatomy including those patterns that have rarely been described. Careful review of preoperative imaging and vigilant intraoperative dissection may prevent injury to these vascular structures and subsequent complications.

## Figures and Tables

**Table 1:** Michels' classification of hepatic arterial anatomy.

Type	Description	Percent
1	Normal	55
2	Replaced LHA from LGA	10
3	Replaced RHA from SMA	11
4	Replaced RHA and LHA	1
5	Accessory LHA	8
6	Accessory RHA	7
7	Accessory RHA and LHA	1
8	Replaced RHA and accessory LHA or replaced LHA and accessory RHA	4
9	CHA from SMA	2.5
10	CHA from LGA	0.5

**Table 2:** Hiatt's classification of hepatic arterial anatomy.

Type	Description	Percent
1	Normal	75.7
2	Replaced or accessory LHA	9.7
3	Replaced or accessory RHA	10.6
4	Replaced or accessory RHA and replaced or accessory LHA	2.3
5	CHA from SMA	1.5
6	CHA from aorta	0.2
